# Immunoglobulin A levels and its correlation with neutrophil-to-lymphocyte ratio as inflammatory biomarkers for dry eye disease in type 2 diabetes: a retrospective study

**DOI:** 10.3389/fimmu.2023.1184862

**Published:** 2023-07-14

**Authors:** Amani Y. Alhalwani, Khulud Abudawood, Al Baraa Ehab A. Qadizadah, Shatha Jambi, Naif S. Sannan

**Affiliations:** ^1^ College of Science and Health Professions, King Saud bin Abdulaziz University for Health Sciences, Jeddah, Saudi Arabia; ^2^ Department of Biomedical Research, King Abdullah International Medical Research Centre, Jeddah, Saudi Arabia; ^3^ College of Nursing, King Saud bin Abdulaziz University for Health Sciences, Jeddah, Saudi Arabia; ^4^ College of Medicine, King Saud bin Abdulaziz University for Health Sciences, Jeddah, Saudi Arabia; ^5^ College of Applied Medical Sciences, King Saud bin Abdulaziz University for Health Sciences, Jeddah, Saudi Arabia

**Keywords:** dry eye disease (DED), type 2 diabetes (T2D), neutrophil-to-lymphocyte ratio (NLR), immunoglobulin a (IgA), inflammation

## Abstract

**Background:**

The neutrophil-to-lymphocyte ratio (NLR) and immunoglobulin A (IgA) level are commonly used as biomarkers for inflammation. Patients with type 2 diabetes (T2D) may experience an imbalance of tear film and inflammation, which can result in dry eye disease (DED). This study aimed to assess the levels of IgA and explore its correlation with the NLR as potential inflammatory biomarkers for dry eye disease in patients with T2D.

**Methods:**

A retrospective study was conducted at the cornea clinic and diabetes centre of King Abdulaziz Medical City (Jeddah, Saudi Arabia). The study included patients with DED and the number of available T2D-DED patients determined the sample size. Neutrophil, lymphocyte, IgA and CRP (C-reactive protein) laboratory values were obtained from medical records and correlational analyses were performed.

**Results:**

The study included 85 patients with an average age of 54 ± 14.4 years for the DED group (n=32) and 62 ± 13.9 years for the T2D-DED group (n=53). The age difference between the two groups was statistically significant (p 0.0001). The NLR values of the T2D-DED and DED groups were 3.203 ± 0.66 and 2.406 ± 0.46, respectively, with no significant difference (p<0.285). Similarly, there were no significant differences in neutrophil and lymphocyte values between the two groups. The IgA levels showed no significant variation between T2D-DED and DED groups (p<0.364). Spearman’s correlation analysis in the DED group showed a significant negative correlation between IgA and lymphocyte (p=0.011; r= - 0.471) values and significant positive correlations between IgA and neutrophil (p=0.014; r=0.309) and NLR (p=0.052; r= - 0.283) values. In the T2D-DED group, a significant correlation was found between IgA and CRP values (p=0.032; r=0.33).

**Conclusion:**

Although diabetic patients may exhibit higher levels of NLR and IgA that correlate with disease severity, our study did not find significant differences in NLR and IgA values between the two groups. These findings may guide future research and enhance understanding of the disease’s underlying mechanisms.

## 1 Introduction

About one in every five adults in Saudi Arabia is affected by diabetes mellitus (DM) and this is expected to double by 2030 (1, 2). Patients with DM may experience microvascular complications such as diabetic retinopathy, nephropathy, neuropathies and diabetic foot (3). Macrovascular complications include severe illnesses such as cerebrovascular, peripheral artery and coronary heart disease. DM may also lead to ocular conditions including glaucoma, retinopathy and DED (4). DED, which is estimated to affect a significant number of urban-dwelling Saudi adults, affects adults over 50 years old (5, 6) and can be classified into two types: aqueous tear deficiency and lipid deficiency evaporative dry eye. The currently available therapies such as eye drops and punctal plugs are mostly palliative and generally ineffective (7). DED is a multifactorial condition that is induced by inflammation and tear film homeostasis imbalance (8). Pan et al. found that people with DM may experience DED due to changes in osmolarity, lacrimal gland dysfunction and altered enzyme metabolism (9). Several studies have indicated that proteins including transferrin, protein kinase C, glucose metabolism proteins, antioxidant proteins and immunoglobulins can be modified by hyperglycaemia, oxidative stress and other consequences of DM (10–12). Individuals with DM also have a different tear protein composition, suggesting a pathological link (13). DED is associated with changes in hormone levels such as 7β-estradiol, estrone and total testosterone. This is an important factor that contributes to the overall clinical presentation of DED (14). DM has also been associated with prolonged epithelial abnormalities, modifications to corneal and conjunctival epithelium and the potential for vision loss due to corneal scarring (15). Corneal and conjunctival epithelial cells express pleiotropic pro-inflammatory cytokines and chemokines (such as tumour necrosis factor, interleukins-1, 6, 8 and nerve growth factor) and matrix-degrading proteases (such as matrix metalloproteinase-9 and matrix metalloproteinase-3) (16–19).

Immunoglobulin A (IgA) is the most prevalent antibody produced in the human body (20) and plays a critical role in protecting and preserving the health of mucosal surfaces, including the eyes, from the harmful effects of inflammatory conditions (21). It is produced in tears and safeguards the eyes from infection. During the inflammatory phase of type 2 diabetes (T2D**)**, the anti-inflammatory properties of IgA are crucial in preventing tissue damage (22). It also stimulates a variety of immune cells expressing the Fc receptor in multiple organs which leads to the production of pro-inflammatory cytokines. The contribution of Fc receptor I-mediated inflammation to the disease remains unclear. The primary immunoglobulin present in tears is IgA, which is a critical immune defence mechanism against infections. Plasma cells in the adenoid or epithelial layer of the conjunctiva produce IgA which is then secreted into the tears in addition to subepithelial cells in the lacrimal gland (23).

Alhalwani conducted a review on the role of neutrophil inflammation in T2D (24). Although the link between T2D and inflammation is well established, few studies have used a reliable biomarker to demonstrate a direct association between T2D and the immune system (25). The NLR serves as a straightforward inflammation biomarker (26, 27). Recent studies have indicated that the NLR, a popular biomarker in both biological and medical research, is associated with numerous chronic inflammatory diseases (24). It is a widely available and cost-effective marker which makes it a commonly used measurement in clinical research. Nonetheless, only a limited number of studies have investigated the relationship between NLR and T2D (28, 29). Our previous study showed that high NLR was positively correlated with CRP in T2D-DED patients compared to DED and T2D patients (28).

In this study, we assessed the levels of NLR and IgA in individuals with DED, with and without T2D, to identify potential inflammatory risk factors for the development of DED. We hypothesised that NLR and IgA could serve as prognostic biomarkers for DED in patients with T2D.

## 2 Methodology

### 2.1 Study population

This study used a retrospective case-control study design with sequential sampling methods, analysing data collected between June 2018 and October 2020. Patients with DED were selected from the outpatient clinic of King Abdulaziz Medical City in Jeddah, Saudi Arabia. Based on the primary indications for DED, they were divided into two groups: those with DED only and those with DED and T2D. The sample size was influenced by the number of available patients who visited the clinic.

Patients under 25 years old, smokers, those wearing contact lenses, those who had refractive surgery and those with other chronic illnesses such as rheumatoid arthritis, lupus, scleroderma, Sjogren’s syndrome, cancer, thyroid disorders, vitamin A deficiency, cardiovascular disease and hypertension were excluded from the study.

### 2.2 Data collection and analysis

Encrypted patient data were obtained from the hospital information system and retrieved using the ICD-10 codes listed in their medical records. Patient information including demographics (age and gender), medical history (including the duration of diabetes - ≤ 4 years and ≥ 4 years, based on the available data in the electronic medical records), presence of diabetic nephropathy, retinopathy, or neuropathy, T2D medication and DED medications) and diagnostic values (blood plasma neutrophils, lymphocytes and blood serum IgA) was collected. The NLR was computed by dividing the neutrophil count (x10^9^/L) by the lymphocyte count (x10^9^/L).

### 2.3 Statistical analysis

PRISM software (GraphPad Inc., San Diego, CA, USA) was used to process and analyse the data. Categorical variables were expressed as percentages and integers. Mean and standard deviation were both used to present the parametric data. Significance between groups was determined for normally distributed data (age, neutrophil, lymphocyte and IgA). For continuously distributed data with anomalous distributions, the Mann-Whitney test was used and for categorical data (by gender), Pearson’s Chi-Square test was used. The correlations between NLR and IgA among DED and T2D-DED patients were examined using Spearman’s correlation tests. A significance level of P <0.05 was adopted.

### 2.4 Ethical consideration

The study obtained ethical approval from the Institutional Review Board at King Abdullah International Medical Research Centre (Jeddah, Saudi Arabia) with the reference number IRB SP20/280/J.

## 3 Results

Of the 85 patients, the T2D-DED group (n=53) consisted of 40% males and 60% females, while the DED group (n=32) included 47% males and 53% females. The average age for the T2D-DED group was 62 ± 13.87 years and for the DED group was 54 ± 14.44 years. The difference in age between the two groups was found to be significant (p=0.0001), while there was no significant difference in the distribution of gender between the two groups (p=0.107; see **Table 1**.

**Table 1:** The demographic data indicators and laboratory findings for T2D-DED and DED.

Parameters	Type 2 diabetes + DED n=53	DED n=32	*P value
Age (years)	62 ± 13.87	54 ± 14.44	0.0001
Gender Male Female	21(40%) 32(60%)	15(47%) 17(53%)	0.107
Neutrophil count x10^9^/L	4.36 ± 2.84	3.71 ± 2.23	0.0776
Lymphocyte count x10^9^/L	4.11 ± 11.58	1.96 ± 0.86	0.228
NLR	3.186 ± 5.08	2.095 ± 1.26	0.285
Immunoglobulin A (g/L)	3.22 ± 2.16	2.72 ± 1.15	0.364
CRP (mg/L)	27.52 ± 53.86	–	NA*
HbA1C (%)	7.34 ± 1.40	–	NA*

*Mann-Whitney test.

CRP (C-reactive protein), HbA1c (Haemoglobin A1c). *CRP and HbA1c data were often not indicated in the medical records of DED patients. Hospital reference intervals: neutrophil 2.0 - 7.5 x 10^9^/L, lymphocyte 1.5 - 4.0 x 10^9^/L, immunoglobulin A 1.01 - 6.45 g/L, CRP <5mg/L, HbA1C <5.7%.

T2D-DED patients had a diabetes duration of ≤ 4 years in 66% of cases and ≥ 4 years in 34% of cases. Of these patients, 90% were controlled with diabetes medication and 48% with DED medication. Additionally, 9% of patients had diabetic nephropathy, 9% had retinopathy and 5% had neuropathy.

Neutrophil and lymphocyte counts for the T2D-DED and DED groups were 4.36 ± 2.84, 4.11 ± 1.58 and 3.71 ± 2.23, 1.96 ± 0.8, respectively. The NLR value was higher in the T2D-DED group compared to the DED group (3.186 ± 5.08 vs 2.095 ± 1.26, respectively) but the difference was not statistically significant (p=0.285). IgA results were 3.222 ± 2.169 and 2.728 ± 1.151 for the T2D-DED and DED groups, respectively, and there was no statistically significant difference in IgA levels between the two groups (p=0.364) (**Table 1**).

**Figure 1:**
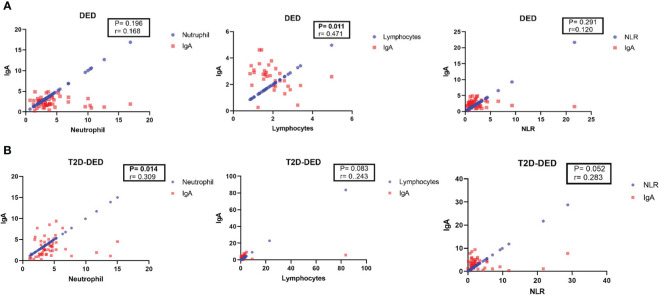
Spearman’s correlation analysis of IgA with neutrophil, lymphocyte and NLR in DED and T2D-DED groups. Scatter plots showing the correlation between IgA and neutrophil, lymphocyte and NLR values in **(A)** DED group and **(B)** T2D-DED group.

In the DED group, Spearman’s correlation analysis revealed no significant correlation between IgA and neutrophil (p=0.196; r= - 0.186) or NLR (p=0.291; r=0.12) values, but a significant negative correlation was found between IgA and lymphocyte (p=0.011; r= - 0.471) values (**Figure 1A**).

In the T2D-DED group, Spearman’s correlation analysis showed no significant correlation between IgA and lymphocyte (p=0.083; r=0.243) values, but significant positive correlations were found between IgA and neutrophil (p=0.014; r=0.309) and NLR (p=0.052; r= - 0.283) values (**Figure 1B**).

**Figure 2:**
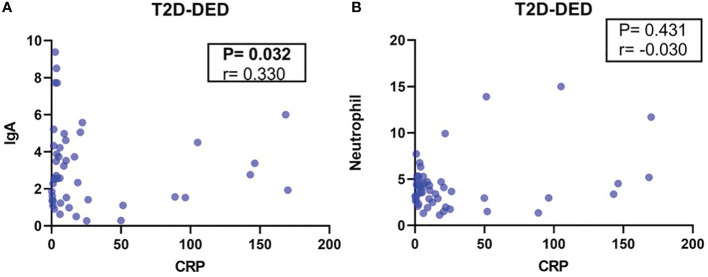
Correlation analysis of IgA, CRP and neutrophil values in the T2D-DED group. Spearman’s correlation analysis was performed to investigate the relationship between IgA and CRP values **(A)** and between CRP and neutrophil values **(B)** in the T2D-DED group.

Spearman’s correlation analysis in the T2D-DED group revealed a significant correlation between IgA and CRP values (p=0.032; r=0.33) (**Figure 2A**), but no significant correlation was found between CRP and neutrophil values (p=0.431; r= - 0.03) (**Figure 2B**). The analysis revealed a significant positive correlation between IgA and CRP values (p=0.032; r=0.33) (A), while no significant correlation was found between CRP and neutrophil values (p=0.431; r=-0.03) (B).

## 4 Discussion

DED is a commonly occurring disorder affecting the ocular surface, with significant implications for vision, quality of life and work productivity (30). Inflammation has been identified as a key pathophysiological mechanism in DED and ocular inflammatory markers have been suggested as potential indicators of DED severity (31). Inflammatory markers such as the concentration of inflammatory mediators in tears and conjunctival cells could be used to assess the severity of DED and may also serve as targets for drug therapy. While anti-inflammatory eye drops have been used to treat severe DED, ocular inflammatory markers have not been used in the diagnosis or prognosis of DED. For research purposes, local inflammatory markers have proven effective in accurately reflecting disease status. However, clinical researchers have encountered difficulties in using tear samples for DED management due to the limited number of samples and challenges in sample collection. The use of immunoassays for inflammatory markers has also been hampered by significant implementation challenges (32).

The present study observed high levels of routine blood measurements including neutrophil, lymphocyte, NLR and IgA in the T2D-DED group as compared to the DED group. Recent studies have highlighted the clinical importance of NLR as new inflammatory indicators derived from the main inflammatory cells, namely neutrophils and lymphocytes in patients with DED. NLR has been widely used to assess the degree of inflammation in conditions such as DM, cardiovascular disease, tumours, autoimmune diseases and inflammatory diseases (33). Studies have also investigated the connections between ocular conditions and NLR, including glaucoma, diabetic retinopathy, age-related macular degeneration, kerato-conjunctivitis and optic neuritis, which often correlates well with disease severity (34–36). The average NLR level in the healthy control subjects reported by Rahar et al. was 1.60 ± 0.18 while in this study the NLR values were higher in both groups (37). Our findings indicate that, although not statistically significant, the NLR value was higher in the T2D-DED group than the DED group (3.186 ± 5.08), which is consistent with other studies with mean results of 3.91 ± 1.68 (37), 2.33 ± 0.15 (28), 2.6 ± 1.2 (38) and 2.8 ± 1.4 (39). This all suggests that the NLR value in DED patients may serve as a potential DED inflammatory indicator in T2D. However, the small sample size used in this study may limit the power of the results and further study with larger sample sizes is needed to confirm the potential use of IgA and NLR as a DED inflammatory indicators in T2D patients.

There are other inflammatory indicators correlated with diabetes such as IgA, which is the most prevalent immunoglobulin isotype on mucosal surfaces and the second most abundant in human plasma (40). IgA is highly correlated with markers of inflammation and fibrinolysis in drug-naive individuals with T2D. Hegde et al. reported an elevation of the IgA level in the serum and saliva of patients with diabetes compared with healthy and non-diabetic patients (41).

Another common inflammatory marker related to diabetes, CRP, has been suggested to be influenced by body fat composition rather than glucose control or insulin sensitivity (42). According to Timpson et al. (2005), metabolic syndrome in women who participated in the British women’s heart and health study was associated with obesity rather than CRP haplotype (43). These results suggest that adipose tissue and its cellular constituents are a significant source of pro-inflammatory cytokine. In contrast, neither HOMA-IR nor HbA1C showed any correlation with CRP levels, indicating that in individuals with DM who were diagnosed within three years and are not taking glucose-lowering medication, systemic inflammation is not influenced by insulin sensitivity or glucose control (43).

Heineke and van Egmond have linked IgA autoantibodies to numerous diseases, including IgA nephropathy (elevated IgA levels), rheumatoid arthritis, coeliac disease and various IgA-associated skin diseases (44).

The association between high levels of IgA and complications in diabetic patients has been previously highlighted (45), implying that monitoring IgA levels could play a vital role in the early detection of potential complications. However, studies investigating the relationship between serum IgA levels and diabetes duration or HbA1C levels found no significant correlation (46, 47). There is also evidence suggesting that elevated NLR levels are linked to elevated HbA1C levels and poor glycaemic control in patients with T2D (48).

Gonzalez-Quintela et al. have previously identified a correlation between serum IgA levels and age. Therefore, we took into account age matching in our analysis (49). We found that the IgA value in the T2D-DED group (3.22 ± 2.16) was higher than that in the DED group (2.72 ± 1.15). This finding aligns with numerous investigations demonstrating that patients with T2D exhibit elevated serum IgA levels compared to healthy subjects, as reported by previous studies (3.50 ± 1.30 (50), 3.41 ± 1.82 (51), and 5.08 ± 1.30 (52)).

This study found that T2D-DED patients had a positive correlation between IgA levels and neutrophil and NLR values. There was also a significant correlation between IgA and CRP values in the same patients. These results suggest that IgA may contribute to the observed systemic inflammation in this population and could be used as a biomarker for inflammation in patients with T2D-DED. Further research is needed on a larger cohort to investigate the mechanisms underlying this association between IgA and inflammation in this context.

One limitation of this retrospective study is that the categorisation of DED was not explicitly documented in the patient database. Rather, clinical diagnosis relied primarily on a physician-administered symptomatic questionnaire which did not provide clear classification or severity information. Future studies should aim to incorporate explicit classification and severity measures to further enhance knowledge in this area.

## 5 Conclusions

NLR is commonly used as an inflammatory biomarker in T2D. However, this study indicates the importance of using NLR for patients with DED. Diabetic complications are frequently linked to higher levels of serum IgA and diabetic patients commonly experience increased circulating IgA levels. Our research indicates that measuring baseline NLR and IgA levels could be beneficial in identifying T2D-DED in the adult population.

## Data availability statement

The original contributions presented in the study are included in the article/supplementary materials. Further inquiries can be directed to the corresponding author.

## Ethics statement

The study obtained ethical approval from the institutional review board at King Abdullah International Medical Research Center (Jeddah, Saudi Arabia) with the reference number IRB SP20/280/J. Written informed consent for participation was not required for this study in accordance with the national legislation and the institutional requirements.

## Author contributions

AA was the study’s principle investigator and the primary researcher who designed the study, supervised the data collection, wrote and approved the final version of this article. KA and NS revised the article and critically analyzed its intellectual, research, and statistical contents. AQ was involved in drafting the article along with data collection and SJ as involved in drafting the article along with data analysis. All authors contributed to the article and approved the submitted version.
